# High Levels of Nutrients of Concern in Baby Foods Available in Europe That Contain Sugar-Contributing Ingredients or Are Ultra-Processed

**DOI:** 10.3390/nu13093105

**Published:** 2021-09-03

**Authors:** Evangelia Grammatikaki, Jan Wollgast, Sandra Caldeira

**Affiliations:** Joint Research Centre (JRC), European Commission, 21207 Ispra, Italy; jan.wollgast@ec.europa.eu (J.W.); sandra.caldeira@ec.europa.eu (S.C.)

**Keywords:** children, complementary feeding, sugars, ultra-processed foods, commercial foods

## Abstract

Introducing children to healthy and diverse complementary foods, either prepared at home or produced commercially, helps to establish taste preferences and good eating habits later in life. Assessing the nutrient profile of foods available commercially is key to informing consumers and policy makers. We used commercial data to provide an overview of the energy and nutrient content of 7 categories of foods intended for infants and young children that were launched or re-launched across 27 European countries from March 2017 to March 2021 (*n* = 3427). We also assessed the presence of sugars as added ingredients, and the foods’ level of processing, using the NOVA classification system. In total, 38.5% of the products contained at least one sugar-contributing ingredient; about 10% of products listed an added sugar, almost ¼ of the products listed a free sugar and finally about 20% of the products listed fruit and vegetable purees and powders as an ingredient. Half of the products had a ‘no added sugars’ positioning statement; among these, almost 35% had free sugars, fruit and vegetable purees and powders as added ingredients. With regard to processing classification, 46.3% of the products were minimally processed, 24.5% were processed and 29.2% ultra-processed. About half of all products had a ‘no artificial ingredient’ positioning statement; however, among these, 31.4% were ultra-processed. Our analysis showed that, within each food category, products with sugars as an added ingredient had a less desirable nutrient profile compared to those that did not have sugar-contributing ingredients. The results for level of processing were similar; in most food categories, ultra-processed foods had higher energy, fat, saturated fat, sugars and sodium content, and lower fibre content, compared to the minimally processed and processed ones.

## 1. Introduction

Providing children with healthy and varied foods from an early age lays the groundwork for their developing taste preferences and for adopting good eating habits throughout adolescence and adulthood [1,2,3]. Exclusive breastfeeding is the preferred and recommended form of nutrition for infants for the first 6 months of life. After the sixth month, ‘children should begin eating safe and adequate complementary foods’ [4] that could be prepared at home or produced commercially [5]. Several countries have developed food-based dietary guidelines (FBDGs) that are specific for older infants and toddlers (i.e., children aged 6 months–3 years) recommending a balanced diet that includes a variety of foods and provides limited amounts of sugars, saturated fat and salt [6]; these nutrients are generally considered nutrients of public health concern across the European region [7,8,9,10,11]. High consumption of sugars has been linked with dental caries and increased body weight from early in life till later in adulthood [12]. Despite this, recent reports [6,13,14] have highlighted high content of total sugars in food products intended for infants and young children (from now on referred to as baby food). In addition, although most FBDGs recommend only limited consumption of fruit juices and sugars at this age [6], the use of concentrated fruit juice, fruit and vegetable (F&V) purees and powders as ingredients in baby food (even in savoury meals) is widespread across Europe [6,14,15]. The pureeing process (both at industrial and home preparations) breaks the F&V cell walls creating readily available free sugars [16].

In the last decades, highly processed packaged foods and drinks have become increasingly available worldwide [17]. Such products are usually energy dense and provide high amounts of sugars, fats and sodium, thus contributing to a higher risk of noncommunicable diseases [17,18]. However, it was difficult to accurately assess the effect such foods had on health since different studies used different definitions. Recently, Monteiro et al. [19] developed the NOVA classification system and, within it, classified foods according to the extent and purpose of industrial processing. Ultra-processed foods (UPFs) were defined as those containing ‘food substances never or rarely used in kitchens … [or] additives designed to make the final product palatable or more appealing’ [19]. The consumption of UPF in childhood has been linked to overweight, diabetes and worse cardiometabolic profiles [20,21,22,23]. The exact causal factors explaining such observations are not yet known, but there is some evidence that higher eating rates observed for diets high in UPF could be a reason for over-consumption [24]. Although several studies have assessed UPF consumption in paediatric populations [25,26,27], there is limited research on the baby food on offer using the NOVA classification system [28].

With our current paper, we first aimed to provide an overview of the energy and nutrient content of baby food launched in European markets from March 2017 to March 2021. Then we wanted to identify within-food-group variations of the nutrient content depending on (a) the presence of sugar-contributing ingredients and (b) the level of processing.

## 2. Materials and Methods

### 2.1. Data Collection

We used the Mintel Global New Products Database (GNPD) to obtain an overview of the baby foods sold in European markets. Each database entry corresponds to a newly (re-)launched product; in case of a relaunched product, the differences can be in the nutrient content, the packaging or the information on the label. We searched the GNPD for all baby foods (re-)launched between March 2017 and March 2021 in 24 EU Member States, Norway, Switzerland and the UK. The retrieved food products were classified into seven categories: baby cereals; baby biscuits and rusks; baby juices and drinks; baby fruit products, desserts and yoghurts; baby snacks; baby savoury meals and dishes; and other baby food (for more details see Appendix A, Table A1). For each of the food products, we retrieved product information, nutrient content, list of ingredients, flavours, allergens and positioning statements.

A total of 4649 products were identified under the category ‘baby food’. As an analysis of milk and follow-on formulas is outside the scope of this paper, these products were excluded from the final analysis (*n* = 894). In addition, some products (*n* = 259) were removed from the analysis when they had incomplete or implausible values for all nutrients examined (for example, missing energy values; more than 100 g of macronutrients in 100 g of product; more than 25 g of carbohydrates or 25 g of protein or 25 g of total sugars in 100 kcal of product; more than 11 g of total or saturated fat in 100 kcal of product; energy values higher than what computed for higher tolerance values for carbohydrates, protein, total fat and fibre; energy values lower than what computed for the lower tolerance values for carbohydrates, protein, total fat and fibre [29]. Furthermore, we removed 16 ‘Baby cereals’ products that were ready-to-eat in jars or pots with no comparable nutrient values to the rest of the products that were in powder form. Similarly, we removed 46 ‘Fruit juices and drinks’ products because the nutrient content provided in the database was for their not ready-to-drink format (for example teabags), and 7 products from the ‘Other baby food’ category that were stock cubes or specialised nutrition products. A total of 3427 baby food products was finally included in the analysis.

### 2.2. Data Analysis

We used the food information data included in the Mintel GNPD on content per 100 g of product for energy (kcal), protein (g), total fats (g), saturated fats (g), carbohydrates (g), total sugars (g), fibre (g) and sodium (mg). For this paper, the term ‘nutrient profile’ will be used to refer to the overall foods’ content in these nutrients; foods that have a higher content in energy or the nutrients of concern (i.e., total and saturated fats, total sugars, sodium) will be referred to as foods with a ‘less desirable nutrient profile’. We also calculated the per 100 kcal values for all examined nutrients.

To assess the use of sugar-contributing ingredients, we went through the ingredient list of the food products included in the final analysis and identified which products contained added sugars [30], free sugars [12], F&V purees and F&V powders. As different definitions and recommendations exist across Europe, we present all analyses separately for products that contain (i) added sugars (as per EU regulation), (ii) free sugars (as per WHO guideline) and (iii) any sugar-contributing ingredient (to cover for countries such as the UK that include F&V purees in free sugars). A variable was calculated to include any food product that contained at least one of the above-mentioned sugar-contributing ingredients.

We further used the list of ingredients to categorise the foods based on the NOVA classification system as follows: minimally processed, processed and ultra-processed [31]. Foods whose ingredients list included only unprocessed foods such as F&V were classified as minimally processed. Products with culinary ingredients such as salt, sugar and fats were classified as processed, while when the ingredient list contained additives, whose function was to enhance flavour, colour or texture, such as flavourings, colourants and emulsifiers, the food product was classified as ultra-processed. Our analysis was based solely on the ingredients listed; when possible to infer from the ingredient name, the use of industrial techniques such as extrusion, hydrogenation and carbohydrate modifications, this was also taken into account.

Additionally, we extracted information from Mintel GNPD on the presence of nutrient (low in…, no added…, high in…, fortified with…, etc.) and natural (organic, free of…, etc.) positioning statements. A ‘no artificial ingredient’ variable was created to include any products that had at least one statement about being free of ‘artificial additives’, ‘artificial colourings’, ‘artificial flavourings’ or ‘artificial preservatives’.

Microsoft Office Excel 365 was used to extract the data and run the quality assurance checks. RStudio with R 4.1.0 was used to analyse the data. The categorical variables were presented as relative and absolute frequencies, and the nutritional information was presented as means and SD. Differences in energy and nutrient content between groups were analysed by the Mann–Whitney test. Differences in the categorical variables across processing classifications or food groups were assessed using the χ^2^ test. Significance was determined at a level of 0.05. Tableau 2020.v4 was used to prepare boxplots to graphically present the distribution of energy content and nutrient density of the products examined (Appendix B).

## 3. Results

We identified 3427 products that were launched or re-launched in the markets of 24 EU Member States, Norway, Switzerland and the UK between March 2017 and March 2021. The majority of new baby food products launched (75.8%) fell under the ‘Baby fruit products, desserts and yoghurts’, ‘Baby savoury meals and dishes’ and ‘Baby cereals’. Appendix A, Table A2 breaks down all products analysed per country and food category.

A description of the nutrient composition of the products is provided in Table 1. ‘Baby biscuits and rusks’ had on average the highest content for energy and all nutrients examined, except for protein and fibre. ‘Baby cereals’ had the highest protein content. ‘Baby snacks’ had the highest fibre content followed closely by ‘Baby cereals’ (mean ± s.d., 4.7 ± 3.1 and 4.6 ± 3.1, respectively).

**Figure 1:**
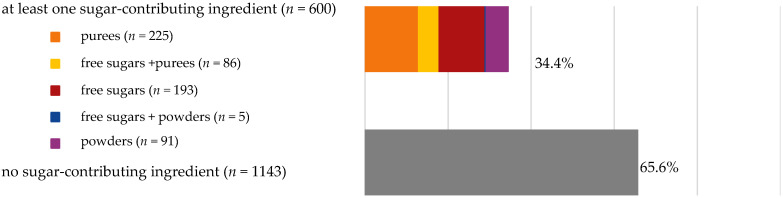
Number of products that made a ‘no added sugar’ claim or statement (*n* = 1743) by presence of sugar-contributing ingredients (free sugars as defined in [12], fruit and vegetable purees, fruit and vegetable powders and combinations thereof).

About 10% of baby foods (*n* = 351) listed an added sugar (as defined in [30]) in their list of ingredients, and almost ¼ of the products (*n* = 835) listed a free sugar (as defined in [12]) (Table 2). About 20% of the products contained F&V purees and F&V powders as an added ingredient (*n* = 510 and *n* = 185, respectively). In total, 38.5% of the products (*n* = 1320) contained at least one sugar-contributing ingredient (Table 2). On the other hand, half of the products had a ‘no added sugars’ positioning statement, most of which were ‘Fruit products, desserts and yoghurts’ and ‘Baby cereals’ (Table 2). Among these, almost 35% (*n* = 600) had free sugars, F&V purees and/or F&V powders as added ingredients (Figure 1).

Overall, products that had added sugars, free sugars or any sugar-contributing ingredient had a less desirable nutrient profile compared to those that did not (Table 3). Products from the ‘Baby cereals’ and ‘Baby biscuits and rusks’ categories with added sugars had higher content for saturated fats, sugars and sodium, and lower fibre content (Table 3). Products from the ‘Baby cereals’ and ‘Baby biscuits and rusks’ categories with added sugars had higher content for saturated fats, sugars and sodium, and lower fibre content (Table 3). Similarly, products with free sugars had lower protein (‘Baby cereals’, ‘Baby biscuits and rusks’), higher saturated fat (‘Baby biscuits and rusks’, ‘Baby fruit products, desserts and yoghurts’ and ‘Baby savoury dishes and meals’), lower fibre (‘Baby cereals’, ‘Baby biscuits and rusks’ and ‘Baby fruit products, desserts and yoghurts’) and higher sodium (‘Baby biscuits and rusks’ and ‘Baby fruit products, desserts and yoghurts’) content (Table 3). When comparing products containing any sugar-contributing ingredient (added or free sugars, F&V purees and F&V powders) with those that did not, the results were again similar. Their energy content was higher in all categories except ‘Baby savoury meals and dishes’; saturated fats content was higher in all categories except ‘Baby snacks’; fibre content was lower in ‘Baby biscuits and rusks’ and ‘Baby fruit products, desserts and yoghurts’; and finally, sodium content was higher in ‘Baby fruit products, desserts and yoghurts’ and ‘Baby snacks’ (Table 3).

**Figure 2:**
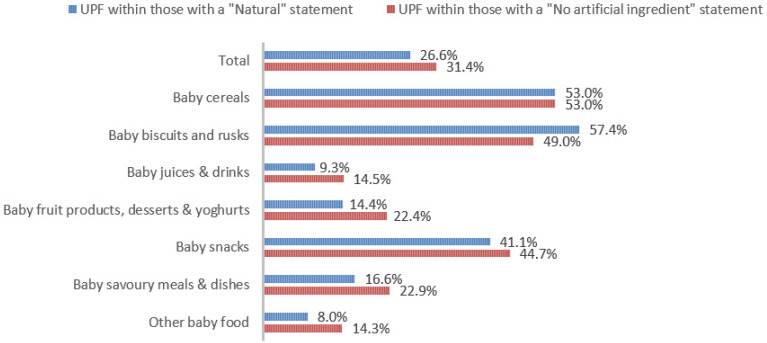
Percentage of ultra-processed foods (UPFs) within products that had a ‘natural’ (*n* = 3093) or ‘no artificial ingredient’ (*n* = 1578) positioning statement. The ‘no artificial ingredient’ category includes all products that had at least one statement about being free of ‘artificial additives’, ‘artificial colourings’, ‘artificial flavourings’ or ‘artificial preservatives’.

With regard to processing classification, 46.3% (*n* = 1586) of the products were minimally processed (Table 2). The highest proportion of minimally processed foods were observed for ‘Baby juices and drinks’ (81.6%), ‘Baby fruit products, desserts and yoghurts’ (73.4%) and ‘Other baby food’ (63.6%) (Table 2). About 30% of the products (*n* = 1001) were UPFs (Table 2). The highest proportion of UPFs was observed for ‘Baby biscuits and rusks’ (62.2%), ‘Baby cereals’ (57.1%) and ‘Baby snacks’ (42.1%) (Table 2). Most of the products (90.3%) had a ‘natural’ positioning statement (Table 2); among these, 26.6% (*n* = 824) were ultra-processed (Figure 2). The highest proportion of products that bore a natural positioning statement and were UPFs were observed in ‘Baby biscuits and rusks’ (57.4%), ‘Baby cereals’ (53.0%) and ‘Baby snacks’ (41.1%) (Figure 2). Similarly, about half of all products had a ‘no artificial ingredient’ positioning statement (Table 2); among these, 31.4% (*n* 496) were UPFs (Figure 2).

Overall, UPFs had a less desirable nutrient profile compared to those with lower levels of processing (Table 4). Compared to the minimally processed and processed foods, UPF had higher energy content (‘Baby cereals’, ‘Baby fruit products, desserts and yoghurts’, ‘Baby snacks’ and ‘Baby savoury meals and dishes’), higher fat content (‘Baby cereals’, ‘Baby juices and drinks’, ‘Baby fruit products, desserts and yoghurts’, ‘Baby snacks’ and ‘Baby savoury meals and dishes’), higher saturated fat content ((‘Baby cereals’, ‘Baby biscuits and rusks’, ‘Baby fruit products, desserts and yoghurts’, and ‘Baby savoury meals and dishes’), higher sugars content (‘Baby cereals’, ‘Baby biscuits and rusks’, ‘Baby fruit products, desserts and yoghurts’ and ‘Baby snacks’), lower fibre content (‘Baby biscuits and rusks’, ‘Baby fruit products, desserts and yoghurts’ and ‘Baby savoury meals and dishes’), and higher sodium content (for all categories except for ‘Baby juices and drinks’ and ‘Other baby food’) (Table 4).

**Figure 3:**
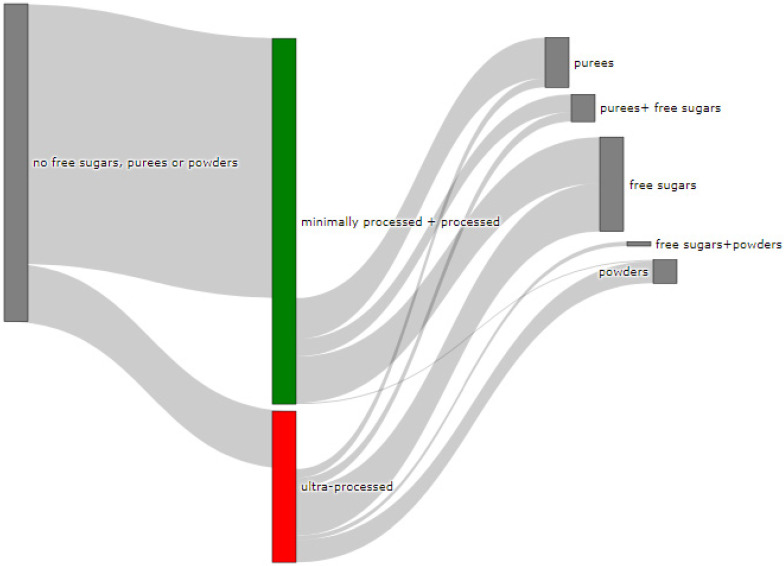
Graphical representation of the proportion of minimally processed/processed foods (green bar, *n* = 2426) and ultra-processed foods (red bar, *n* = 1001) that contained free sugars (as defined in [12]), fruit and vegetable purees, and fruit and vegetable powders as an ingredient (to the right) or did not contain any of these sugar-contributing ingredients (to the left). The graph is read starting from the middle (coloured bars). The length of the bars is based on the number of products within each category and indicates the proportion in the total sample.

Finally, Figure 3 shows what proportion of minimally processed/processed and UPFs had free sugars, F&V purees or F&V powders as an added ingredient. More than 28% of the minimally processed/processed foods (*n* = 696) contained F&V purees and/or free sugars (mainly fruit juices or fruit juice concentrates). On the other hand, more than 60% of UPFs contained at least one sugar-contributing ingredient; the majority contained free sugars and/or F&V powders (combined *n* = 558).

## 4. Discussion

This paper used commercially available food composition data, to provide an overview of the energy and nutrient content of baby food launched or re-launched in the markets of 27 European countries from March 2017 to March 2021 (*n* = 3427). It also identified within-food-group variations of the energy and nutrient content depending on (a) the presence of sugar-contributing ingredients and (b) the level of processing (using the NOVA classification system).

According to the European Food Safety Agency recommendations [32], the average requirements for energy for older infants and toddlers range from 573 (for 7-month-old girls) to 1170 kcal (for 3-year-old boys) per day. Some of the food categories had relatively high energy content; 100 g of ‘Baby biscuits and rusks’, ‘Baby cereals’, ‘Baby snacks’ or ‘Other baby food’ could contribute from ¼ to ¾ of the daily energy requirements. If consumed in excess, they could either displace other important more nutrient-dense food categories or lead to energy overconsumption and unfavourable gain in body mass [33].

The highest content of total sugars was observed for ‘Baby biscuits and rusks’, ‘Baby snacks’ and ‘Baby cereals’ (16.1, 15.4 and 14.8 g per 100 g of product, respectively). These are quite high if one takes into account the guideline to limit free sugar intake to 5% or 10% of the overall energy intake [12] and the EFSA Average Requirements for energy [32] for infants and young children (Table 5). García et al. [15] found high sugar content in fruit snacks, cereal bars, and cereals (mean content: 22.9, 48.4 and 28.9 g, respectively). Hilbig et al. [34] found the highest contents of added sugars in commercial dairy–fruit meals. Similarly, Rito et al. [35] found that infant cereals had the highest total sugar content of all ready-to-eat cereals in Portugal. Marinho et al. [36] identified the main dietary sources of sugar intake in children <5years old in Portugal; 69% of added- and 67% of free sugar intake were from yoghurts, infant cereals, infant formula, sweets and cookies. Devenish et al. [37] examined free sugar intakes in a sample of 2 year olds in Australia; the main sources were non-core foods, such as fruit juice, biscuits, cakes, desserts and confectionery.

**Figure A1:**
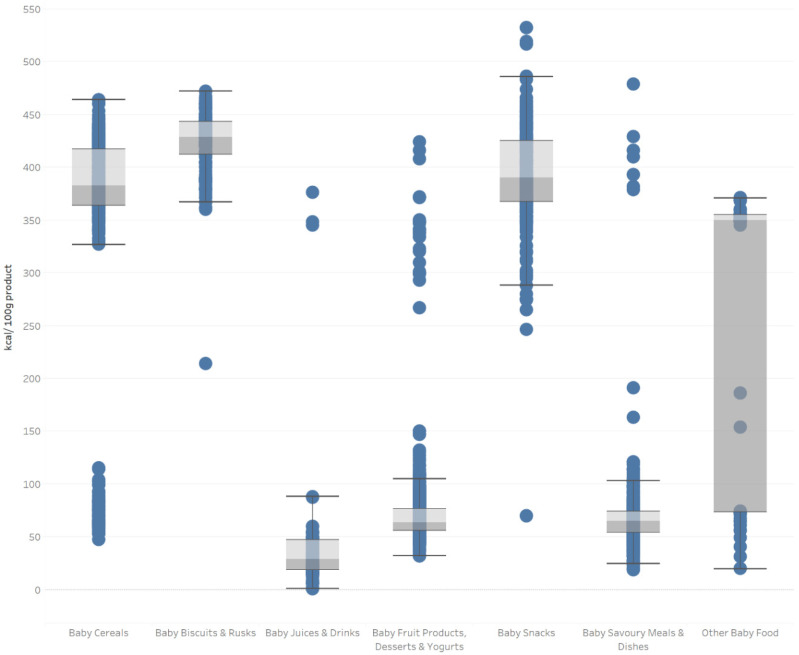
Energy content (kcal/100 g product) by baby food category.

**Figure A2:**
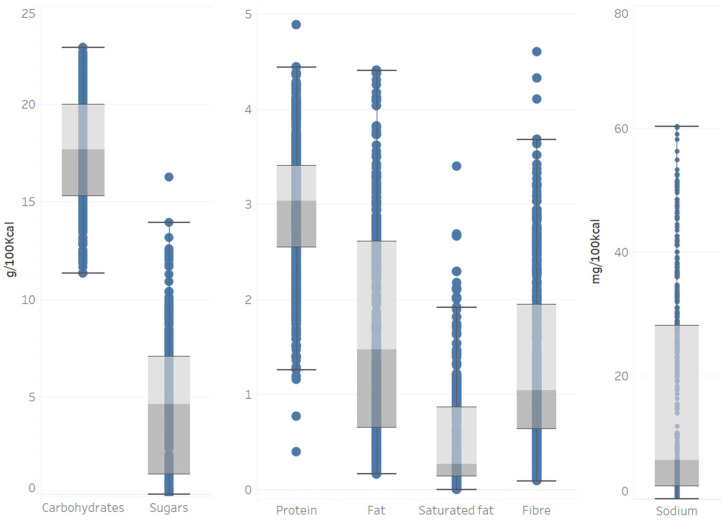
Nutrient content (g/100 kcal product, except for sodium mg/100 kcal product) of ‘Baby cereals’.

**Figure A3:**
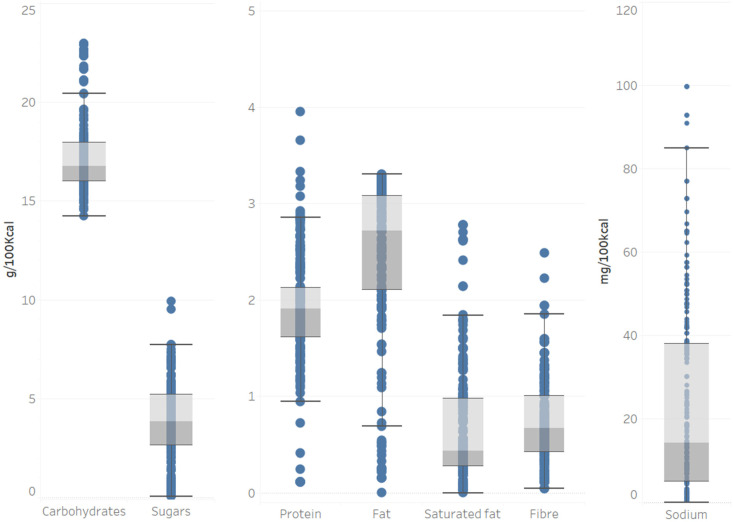
Nutrient content (g/100 kcal product, except for sodium mg/100 kcal product) of ‘Baby biscuits and rusks’.

**Figure A4:**
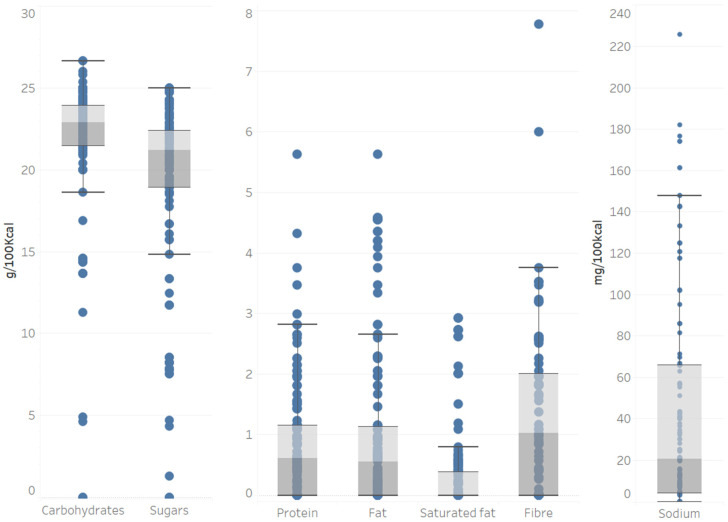
Nutrient content (g/100 kcal product, except for sodium mg/100 kcal product) of ‘Baby juices and drinks’.

**Figure A5:**
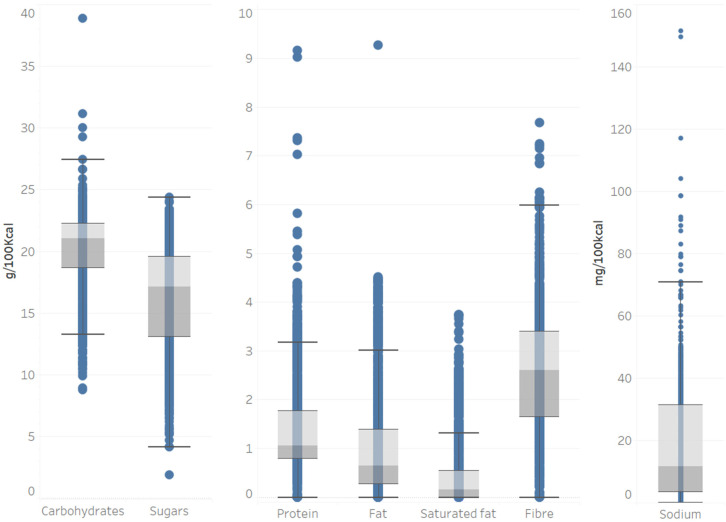
Nutrient content (g/100 kcal product, except for sodium mg/100 kcal product) of ‘Baby fruit products, desserts and yoghurts’.

**Figure A6:**
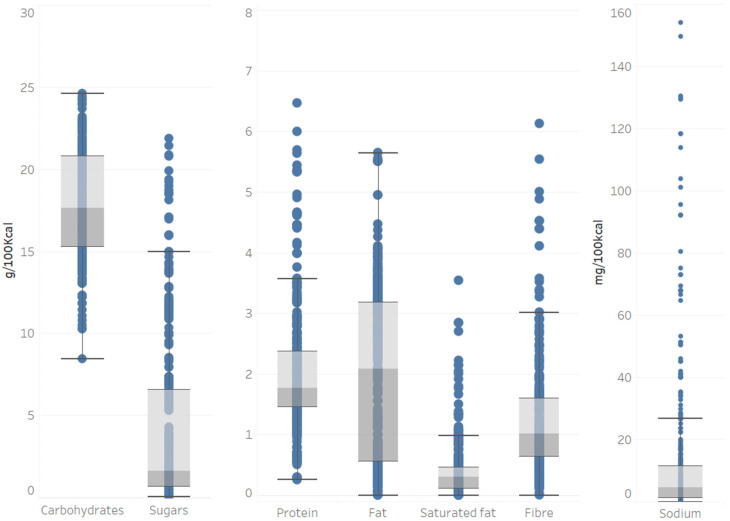
Nutrient content (g/100 kcal product, except for sodium mg/100 kcal product) of ‘Baby snacks’.

**Figure A7:**
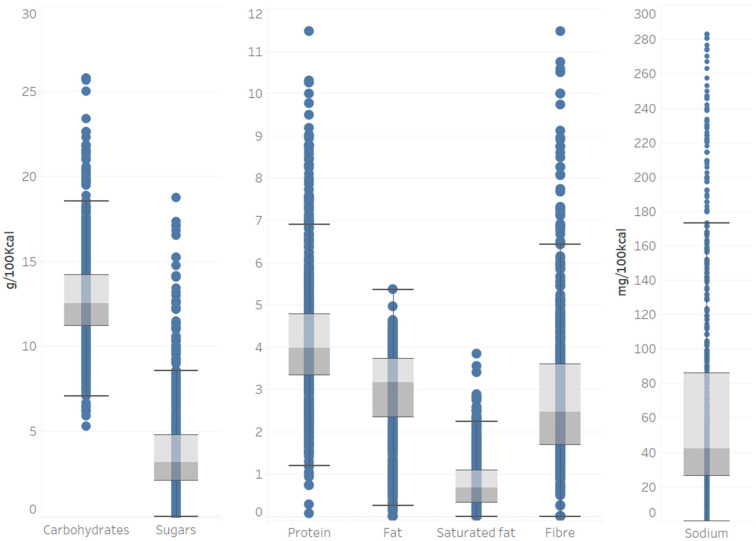
Nutrient content (g/100 kcal product, except for sodium mg/100 kcal product) of ‘Baby savoury meals and dishes’.

**Figure A8:**
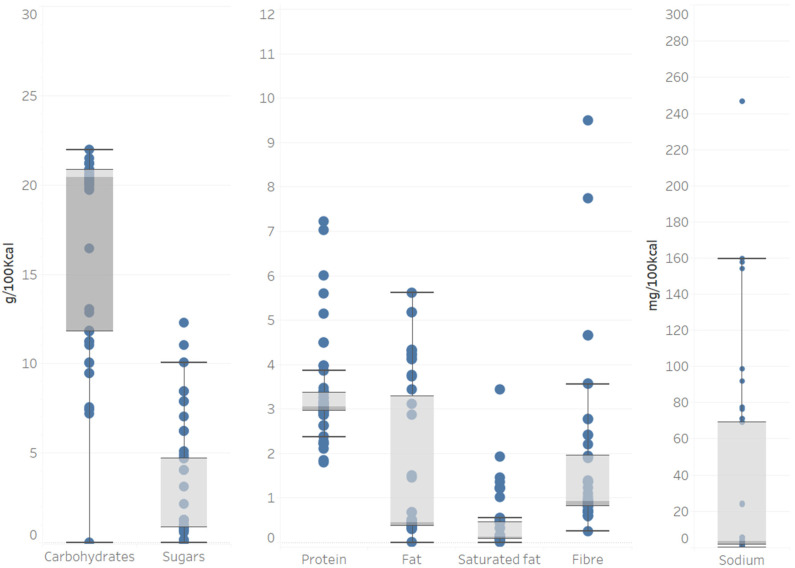
Nutrient content (g/100 kcal product, except for sodium mg/100 kcal product) of ‘Other baby food’.

The energy and nutrient content by food category in this paper are presented per 100 g (Table 1) or per 100 kcal of a product (Figure A1, Figure A2, Figure A3, Figure A4, Figure A5, Figure A6, Figure A7 and Figure A8) for comparability with other studies; however, children may consume less at a single eating occasion or throughout the day, also depending on their age. Table 5 provides an easy reference point for parents and health professionals who want to quickly compare the energy or sugar content of the consumed portion or overall diet with the current recommendations.

In our sample, 38.5% of the products contained at least one sugar-contributing ingredient (added sugars, free sugars, F&V purees or F&V powders). Almost ¼ of the products listed a free sugar (as defined in [12]) as an ingredient. Hutchinson et al. [14] reported that on average, between 21% (Denmark) to 58% (Hungary) of products listed an ‘added sugar’ (with a definition similar to ‘free sugars’ by [12]) as an ingredient. In the current study, 57.1% of ‘Baby biscuits and rusks’ and 29% of ‘Baby fruit products, desserts and yoghurts’ contained free sugars. Although the food categories do not correspond exactly, these findings are in line with those by Hutchinson et al. [14] that found high levels of free sugars in ‘fruit purees (with cereal or milk)’ and ‘dry cereals (with high-protein foods)’. A German study that assessed commercial and home-made meals of 6–12-month-old infants found added sugars in less than a quarter of the meals [34].

Furthermore, in the current study, half of the products (*n* = 1743) had a ‘no added sugars’ positioning statement. More than 1/3 of those bearing such a statement included free sugars, F&V purees and/or F&V powders as added ingredients. Similarly, García et al. [15] found that of the products that had a ‘no added sugar’ claim, half contained concentrated fruit juice, fruit puree or both. Processed fruit (pureed or in the form of smoothies) are generally considered by public as healthier to added or free sugars; however the pureeing process breaks down the F&V cellular structure and the released sugars seem to have the same effect as other forms of sugar [16].

The current study found that about 30% of the baby food products were UPFs. There is limited research on the baby food on offer using the NOVA classification system. Rocha et al. [28] ran such study in Brazil, collecting data in-store, and found 79% of the foods were UPFs. This higher proportion can be explained by the fact that in their analysis, they also included breast milk substitutes and follow-up formulas (33% of the sample), which in the NOVA classification system are classified as ultra-processed due to the manufacturing process.

We also showed that out of all UPFs that contained at least one sugar-contributing ingredient (*n* = 617/1001), the vast majority (90%) contained free sugars or F&V powders, and about 20% contained F&V purees. On the other hand, out of the minimally processed/processed foods that contained sugar-contributing ingredients (*n* = 703/2426), more than half (55%) contained F&V purees, 60% contained free sugars (mainly fruit juices or fruit juice concentrates) and 1% contained F&V powders. Although a few studies have examined the sugar content by level of processing [26,28,38], we are not aware of another study that has tried to identify the type of the main sugar-contributing ingredients by level of processing in baby food.

Our study also found a less desirable nutrient profile for foods that had sugars as added ingredients or were ultra-processed. Regarding the nutrient profile by level of processing, our findings are concordant with those of other studies from Brazil and Canada [26,28,38,39] reporting that UPFs consumed by the population had higher energy, fat, free sugars and sodium content, and lower fibre content compared with less processed foods.

The intake of both sugars and of UPFs impacts health parameters both at childhood and later in life [12,18,20,21,22,23]. In addition, adopting dietary patterns high in foods with sugars as added ingredients or high in UPFs, already from an early age, may affect children’s appetitive traits and increase their preference for highly palatable foods that are rich in sugars, fats or sodium throughout their life [1,2]. Many researchers, scientific organisations and health professional associations have highlighted the importance of addressing sugar intake and UPF consumption in childhood to alleviate the multi-faceted burden of childhood obesity [12,25,40]. Policies targeting these factors can also help to reduce health inequalities since disadvantaged children coming from low-income households are more likely to consume diets higher in free sugars or UPFs [25,37].

Our study has several limitations and strengths. We obtained a large number of food products from across Europe. Nutritional data were obtained from a commercial database and although we could not validate the content of food labels ourselves, we performed a series of quality assurance checks (as described in the Section 2, and checked the pictures of the labels that were available from Mintel GNPD, in the case of outlying or extremely low/high values). We used the food categories as defined within Mintel GNPD to ensure comparability to past or future studies using the same commercial data provider. The labelling of micronutrient content is not mandatory for baby food in the EU, and we could not obtain data on these important nutrients for the products analysed. Our results are not sales-weighted, meaning we could not assess children’s overall intakes or consumption of specific foods/food groups. We used the ingredients listed in the Mintel GNPD to classify the foods products according to their level of processing; we could not validate that all ingredients were correctly input from the labels or assure the accuracy of the translations of the ingredient lists. Despite these limitations, our analysis regarding the NOVA classification is the first to be done in such a big sample of baby foods and can add to the limited research in the area.

## 5. Conclusions

The current study provides information on the energy and nutrient content of foods that are intended for infants and young children in the European region. Using a commercial database, our analysis shows high proportions of baby foods that include sugars as an added ingredient or that are UPFs. We also showed that such products have less desirable nutrient profiles with high contents of fat, saturated fats, sugars and sodium, and low fibre content. Our findings highlight the importance of promoting—for example, through FBDGs—the consumption of minimally processed foods with low added- or free sugar content, such as wholegrain cereals, legumes, whole fruits and vegetables to infants and young children. In addition, policies need to be strengthened to guide product reformulation and improve nutrient profiles of products, while also taking into account the level of processing.

## Figures and Tables

**Table 1:** Energy and nutrient content (mean ± s.d.) per 100 g of 3427 foods for infants and young children from 24 EU countries, Norway, Switzerland and the UK.

	Baby Cereals (*n* = 571)	Baby Biscuits and Rusks (*n* = 233)	Baby Juices and Drinks (*n* = 147)	Baby Fruit Products, Dessertsand Yoghurts (*n* = 1306)	Baby Snacks (*n* = 394)	Baby Savoury Meals and Dishes (*n* = 721)	Other Baby Food (*n* = 55)
Energy (kcal)	351.4 ± 112	423.4 ± 28.8	38.1 ± 49	72.7 ± 43.8	391.2 ± 48.3	68.8 ± 42.6	262.5 ± 136.4
Protein (g)	10.5 ± 4.3	7.9 ± 2.5	0.3 ± 0.4	1.1 ± 1.2	8 ± 4.1	3 ± 2.9	8.6 ± 5
Total fat (g)	5.6 ± 4.4	10.5 ± 3.9	0.3 ± 0.5	0.9 ± 1.2	8.2 ± 6.2	2 ± 1.3	1.9 ± 1.1
Saturated fat (g)	1.6 ± 1.7	3.1 ± 2.8	0.1 ± 0.3	0.4 ± 0.7	1.6 ± 2	0.6 ± 0.6	0.5 ± 0.5
Carbohydrate (g)	62.7 ± 21.5	72.8 ± 6.5	8.4 ± 12.2	14.2 ± 8.5	69 ± 10.1	8.9 ± 7	51.4 ± 31.8
Total sugars (g)	14.8 ± 12.7	16.1 ± 8.4	7.7 ± 12.2	11.3 ± 7.2	15.4 ± 17.5	2.3 ± 1.6	3.3 ± 1.5
Fibre (g)	4.6 ± 3.1	3 ± 1.8	0.4 ± 0.5	1.8 ± 1.9	4.7 ± 3.1	1.8 ± 1.2	2.7 ± 1
Sodium (mg)	52.5 ± 60.5	100.9 ± 99.9	14.3 ± 25	14.4 ± 29.6	55.8 ± 111.5	46.1 ± 51.7	30.4 ± 44.4

**Table 2:** Description of food products according to presence of sugar-contributing ingredients, NOVA classification system score and presence of positioning statements.

		All Products (*n* = 3427)	Baby Cereals (*n* = 571)	Baby Biscuits and Rusks (*n* = 233)	Baby Juices and Drinks (*n* = 147)	Baby Fruit Products, Desserts and Yoghurts (*n* = 1306)	Baby Snacks (*n* = 394)	Baby Savoury Meals and Dishes (*n* = 721)	Other Baby Food (*n* = 55)
Ingredients	Added sugars ^1a^ Free sugars ^2a^ Purees ^a^ Powders ^a^ Any sugar-contributing ingredient ^a^	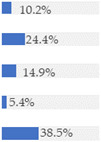	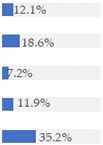	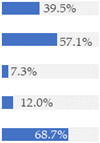	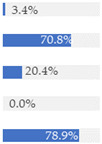	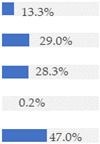	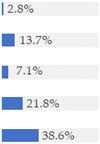	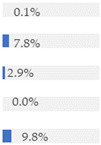	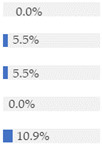
NOVA classification ^a^	Minimally processed	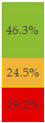	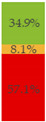	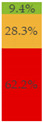	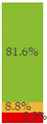	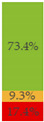	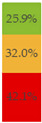	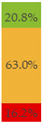	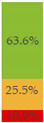
	Processed								
	Ultra-processed								
Positioning statements	Nutrient-related ^a^ ‘No added sugar’ ^a^ ‘Natural’ ^a^ ‘Free of artificial ingredients’ ^3^	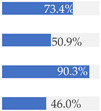	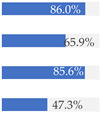	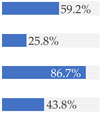	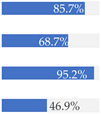	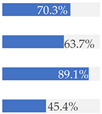	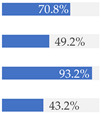	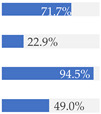	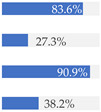

^1^ As defined in [30]; ^2^ as defined in [12]; ^3^ this includes products that had at least one statement about being free of ‘artificial additives’, ‘artificial colourings’, ‘artificial flavourings’ or ‘artificial preservatives’; ^a^ <0.001.

**Table 3:** Comparison of energy and nutrient content (mean ± s.d.) of products that did or did not contain added sugars, free sugars or any sugar-contributing ingredient, by baby food subcategory.

		*n*	Energy	Protein	Fat	Saturated Fats	Carbohydrates	Sugars	Fibre	Sodium
			kcal/100 g	g/100 g	g/100 g	g/100 g	g/100 g	g/100 g	g/100 g	mg/100 g
Added Sugars										
Baby cereals	No	502	353.77 ± 107.98	10.57 ± 4.27	5.53 ± 4.44	1.56 ± 1.61	63.11 ± 20.73	13.7 ± 12.41	4.82 ± 3.12	50.16 ± 60.6
	Yes	69	333.83 ± 137.65	9.77 ± 4.57	5.72 ± 3.93	2.19 ± 1.93 ^b^	59.55 ± 26.32	22.8 ± 12.07 ^c^	2.64 ± 2.14 ^c^	69.59 ± 57.61 ^c^
Baby biscuits and rusks	No	141	416.55 ± 31.01	8.52 ± 2.1	10.03 ± 4.53	2.43 ± 2.05	71.28 ± 7.33	12.33 ± 7.4	3.52 ± 1.9	92.69 ± 102.55
	Yes	92	433.79 ± 21.47 ^c^	6.99 ± 2.87 ^b^	11.12 ± 2.73	4.02 ± 3.38 ^c^	75.09 ± 4.22 ^c^	21.92 ± 6.29 ^c^	2.29 ± 1.34^c^	113.52 ± 94.81 ^b^
Baby fruit products, desserts and yoghurts	No	1133	69.85 ± 43.86	0.92 ± 1.02	0.67 ± 1.03	0.27 ± 0.57	14.16 ± 8.74	11.45 ± 7.55	1.96 ± 1.93	11.86 ± 30.09
	Yes	173	91.62 ± 38.19 ^c^	2.42 ± 1.51 ^c^	2.39 ± 1.4 ^c^	1.43 ± 0.83 ^c^	14.76 ± 6.91 ^c^	10.58 ± 4.22 ^b^	0.73 ± 0.53 ^c^	31.01 ± 19.17 ^c^
Baby snacks	No	383	392.26 ± 44.64	8.00 ± 4.04	8.27 ± 6.1	1.59 ± 1.97	69.13 ± 9.55	15.36 ± 17.67	4.72 ± 3.1	54.03 ± 108.88
	Yes	11	355.97 ± 119.45	6.23 ± 4.37 ^a^	7 ± 9.53	2.35 ± 3.58	64.38 ± 21.54	15.76 ± 8.18	4.57 ± 2.54	117.7 ± 177.65
**Free Sugars**										
Baby cereals	No	465	361.87 ± 99.57	10.83 ± 4.06	5.72 ± 4.5	1.6 ± 1.62	64.4 ± 19.32	13.75 ± 12.48	4.89 ± 3.13	51.54 ± 61.33
	Yes	106	305.29 ± 147.28	8.88 ± 4.99 ^c^	4.83 ± 3.75	1.79 ± 1.82	55.13 ± 28.01	19.42 ± 12.74 ^c^	3.02 ± 2.43 ^c^	56.78 ± 56.99
Baby biscuits and rusks	No	100	413.15 ± 32.54	8.86 ± 2.18	9.28 ± 4.76	2.09 ± 1.78	71.75 ± 8.1	11.27 ± 7.84	3.73 ± 2.04	91.76 ± 110.31
	Yes	133	431.03 ± 23.03 ^c^	7.21 ± 2.56 ^c^	11.35 ± 2.92 ^b^	3.79 ± 3.13 ^c^	73.56 ± 4.97 ^b^	19.83 ± 6.81 ^c^	2.46 ± 1.34 ^c^	107.76 ± 91.06 ^b^
Baby fruit products, desserts and yoghurts	No	927	70.35 ± 48.04	0.93 ± 1.1	0.67 ± 1.05	0.28 ± 0.58	14.22 ± 9.59	11.61 ± 8.25	2.07 ± 2.12	12.33 ± 32.6
	Yes	379	78.58 ± 30.14 ^c^	1.57 ± 1.35 ^c^	1.43 ± 1.46 ^c^	0.78 ± 0.89 ^c^	14.29 ± 5.04 ^c^	10.68 ± 3.42	1.2 ± 0.68 ^c^	19.47 ± 19.7 ^c^
Baby snacks	No	340	392.51 ± 45.17	7.99 ± 4.07	8.33 ± 5.97	1.49 ± 1.59	68.97 ± 9.19	14.28 ± 17.4	4.68 ± 2.99	52.56 ± 106.99
	Yes	54	383.32 ± 64.75	7.71 ± 3.95	7.64 ± 7.57	2.36 ± 3.68	69.22 ± 14.44	22.19 ± 16.48 ^c^	4.96 ± 3.69	76.65 ± 136.39
Baby savoury meals and dishes	No	665	68.71 ± 44.03	2.99 ± 3	2.03 ± 1.3	0.58 ± 0.64	8.84 ± 7.22	2.33 ± 1.58	1.78 ± 1.19	45.55 ± 52.07
	Yes	56	69.93 ± 20.08 ^a^	3.03 ± 1.51	2.03 ± 1.2	0.74 ± 0.64 ^a^	8.98 ± 3.00	2.51 ± 2.05	1.68 ± 0.78	52.29 ± 47.35
**Any Sugar-Contributing Ingredient**										
Baby cereals	No	370	363.18 ± 99.05	10.85 ± 4.11	5.51 ± 4.61	1.51 ± 1.57	65.29 ± 19.65	13.15 ± 12.48	4.65 ± 3.04	51.99 ± 60.11
	Yes	201	329.61 ± 130.14 ^a^	9.77 ± 4.59 ^a^	5.64 ± 3.93	1.88 ± 1.8 ^a^	57.87 ± 23.82 ^c^	17.83 ± 12.61 ^c^	4.38 ± 3.19	53.49 ± 61.43
Baby biscuits and rusks	No	73	411.03 ± 34.32	9.08 ± 2.35	9.31 ± 4.6	1.97 ± 1.65	70.92 ± 8.13	11.17 ± 8.03	4.00 ± 2.02	108.33 ± 119.25
	Yes	160	428.98 ± 24.07 ^c^	7.38 ± 2.45 ^c^	10.99 ± 3.5 ^a^	3.56 ± 3.02 ^c^	73.63 ± 5.5 ^c^	18.42 ± 7.57 ^c^	2.57 ± 1.49 ^c^	97.43 ± 89.85
Baby fruit products, desserts and yoghurts	No	692	71.49 ± 52.59	0.92 ± 1.11	0.66 ± 1.11	0.26 ± 0.58	14.47 ± 10.41	11.89 ± 8.86	2.25 ± 2.45	11.7 ± 21.23
	Yes	614	74.15 ± 30.91 ^c^	1.35 ± 1.28 ^c^	1.16 ± 1.3 ^c^	0.62 ± 0.82 ^c^	13.98 ± 5.68 ^c^	10.72 ± 4.6	1.36 ± 0.7 ^c^	17.47 ± 36.61 ^c^
Baby snacks	No	242	386.9 ± 47.86	8.02 ± 4.47	7.6 ± 6.13	1.47 ± 1.66	68.96 ± 9.63	14.74 ± 17.83	4.87 ± 3.22	42.53 ± 88.91
	Yes	152	398.18 ± 48.42 ^b^	7.85 ± 3.29	9.25 ± 6.2 ^a^	1.84 ± 2.49	69.07 ± 10.72	16.35 ± 16.9 ^c^	4.49 ± 2.86	76.95 ± 137.9 ^c^
Baby savoury meals and dishes	No	650	68.78 ± 44.42	3 ± 3.03	2.03 ± 1.31	0.57 ± 0.64	8.84 ± 7.29	2.32 ± 1.58	1.78 ± 1.2	45.34 ± 51.8
	Yes	71	69.08 ± 20.17	2.94 ± 1.4	2 ± 1.15	0.73 ± 0.61 ^a^	8.93 ± 2.94 ^a^	2.6 ± 1.92	1.67 ± 0.75	52.86 ± 50.84

‘Baby fruit juices and drinks’ is not presented as by definition fruit juices and fruit juice concentrates are considered free sugars; ‘Other baby food’ is not presented due to very few cases; ‘Baby savoury dishes and meals’ is not presented in the analysis by presence of added sugars due to very few cases; nonparametric test Mann–Whitney U was used to compare the mean energy and nutrient values within the same baby food subcategory; ^a^ *p* < 0.05; ^b^ *p* < 0.01; ^c^ <0.001.

**Table 4:** Comparison of energy and nutrient content (mean ± s.d.) between minimally processed/processed foods and ultra-processed foods, by baby food subcategory.

		*n*	Energy	Protein	Fat	Saturated Fats	Carbohydrates	Sugars	Fibre	Sodium
			kcal/100 g	g/100 g	g/100 g	g/100 g	g/100 g	g/100 g	g/100 g	mg/100 g
Baby cereals	(M)PF	245	304.18 ± 131.36	8.44 ± 4.17	3.08 ± 2.39	0.68 ± 0.64	58.33 ± 27.16	5.43 ± 6.13	4.96 ± 3.58	17.29 ± 27.98
	UPF	326	386.82 ± 78.22 ^c^	12 ± 3.76 ^c^	7.41 ± 4.62 ^c^	2.35 ± 1.83 ^c^	65.95 ± 15.19	21.92 ± 11.77 ^c^	4.29 ± 2.71	78.86 ± 64.81 ^c^
Baby biscuits and rusks	(M)PF	88	418.38 ± 34.81	8.71 ± 2.3	9.44 ± 5.08	2.2 ± 2.05	72.91 ± 8.08	12.45 ± 8.09	3.42 ± 2	92.82 ± 110.27
	UPF	145	426.37 ± 24.17	7.43 ± 2.56 ^b^	11.08 ± 2.91	3.58 ± 3.01 ^c^	72.71 ± 5.45	18.39 ± 7.8 ^c^	2.78 ± 1.62 ^a^	105.77 ± 93.06 ^a^
Baby juices and drinks	(M)PF	133	36.23 ± 41.51	0.29 ± 0.37	0.28 ± 0.41	0.11 ± 0.27	8.07 ± 10.27	7.32 ± 10.44	0.43 ± 0.52	14.66 ± 25.58
	UPF	14	55.83 ± 95.09	0.3 ± 0.68	0.4 ± 0.86 ^a^	0.13 ± 0.35	12.01 ± 24.02	11 ± 23.12	0.63 ± 0.12	9.68 ± 16.35
Baby fruit products, desserts and yoghurts	(M)PF	1079	69.43 ± 43.2	0.88 ± 0.91	0.63 ± 0.97	0.25 ± 0.55	14.18 ± 8.69	11.57 ± 7.63	2 ± 1.94	11.52 ± 30.46
	UPF	227	88.45 ± 43.09 ^c^	2.27 ± 1.7 ^c^	2.17 ± 1.5 ^c^	1.26 ± 0.86 ^c^	14.51 ± 7.68 ^c^	10.24 ± 4.51 ^c^	0.91 ± 1.05 ^c^	28.07 ± 20.16 ^c^
Baby snacks	(M)PF	228	387.69 ± 39.7	8.38 ± 4.39	7.35 ± 5.82	1.49 ± 1.82	69.23 ± 10.5	14.48 ± 17.74	5.01 ± 3.44	41.22 ± 92.88
	UPF	166	396.13 ± 57.9 ^b^	7.36 ± 3.47	9.45 ± 6.52 ^a^	1.78 ± 2.27	68.69 ± 9.43	16.58 ± 17.07 ^b^	4.35 ± 2.53	75.62 ± 130.41 ^c^
Baby savoury meals and dishes	(M)PF	604	65.63 ± 32.15	2.93 ± 2.93	1.96 ± 1.31	0.57 ± 0.65	8.27 ± 4.2	2.28 ± 1.49	1.83 ± 1.18	34.7 ± 34.03
	UPF	117	85.23 ± 74.77 ^c^	3.35 ± 2.79	2.4 ± 1.13 ^c^	0.67 ± 0.62 ^b^	11.87 ± 14.14 ^c^	2.71 ± 2.12	1.47 ± 0.99 ^c^	104.71 ± 80.2 ^c^
Other baby food	(M)PF	49	263.13 ± 135.73	8.86 ± 5.06	1.9 ± 1.18	0.46 ± 0.41	51.26 ± 31.81	3.45 ± 1.49	2.76 ± 0.84	29 ± 42.18
	UPF	6	257.53 ± 154.76	6.46 ± 4.51	1.99 ± 0.54	0.78 ± 0.75	52.27 ± 34.77	2.25 ± 1.65	2.36 ± 1.81	41.18 ± 63.31

(M)PF, minimally processed/processed food; UPF, ultra-processed food; nonparametric test Mann–Whitney U was used to compare the mean energy and nutrient density values of ultra-processed foods vs. minimally processed and processed foods within the same baby food subcategory; ^a^ *p* < 0.05; ^b^ *p* < 0.01; ^c^ <0.0014.

**Table 5:** Recommended maximum free sugar intake based on a threshold of 5% or 10% of overall energy intake [12] and the EFSA Average Requirements for energy [32] for infants and toddlers.

Age	Average Requirements for Energy (kcal/day) ^1^	Recommended Maximum Free Sugars Intake (g)	
		5% Energy Intake	10% Energy Intake
7 months	609	7.6	15.2
8 months	633	7.9	15.8
9 months	657	8.2	16.4
10 months	681	8.5	17.0
11 months	705	8.8	17.6
1 year	752	9.4	18.8
2 years	991	12.4	24.8
3 years	1134	14.2	28.4

Table reproduced from [6]; ^1^ average values estimated for males and females.

## Data Availability

Restrictions apply to the availability of these data. Data were obtained from Mintel GNPD.

## Appendix A

This appendix provides information on the foods included under each food category (Table A1) and provides details on the number of products for which information was collected by country and food category (Table A2).

**Table A1:** “Baby food” categories as defined in the Mintel GNPD.

**Baby Cereals**
These products are often a baby’s first introduction to solid food and are commonly in powdered format, but may also be ready to eat in jars or pots. Includes semolina, porridges and creamed rice. Breakfast cereals marketed at babies, such as corn flakes for babies are also categorised here.
**Baby Biscuits and Rusks**
All biscuits, rusks and crackers positioned for babies and toddlers.
**Baby Juices and Drinks**
Beverages for babies in all formats (including beverage mixes and concentrates). Includes fruit juices and fruit and cereal drinks, as well as drinks that claim to also be a meal. Milk drinks and formulas are not included here.
**Baby Fruit Products, Desserts and Yoghurts**
Products in this subcategory include single-fruit purées, multi-fruit purées, fruit and cereal combinations, milky desserts, yoghurts as well as fruit pieces specified for babies and toddlers. Fruit-flavoured snacks are categorised under Baby Snacks.
**Baby Snacks**
Items positioned as snacks for babies belong under this subcategory unless they are items that call themselves snacks but are actually puddings, biscuits, etc.
**Baby Savoury Meals and Dishes**
These products range from vegetable purées, soups, meat preparations, mixed vegetable purées, vegetarian menus (complete meals), complete vegetable meals with meat or fish and all other complete meals.
**Other Baby Food**
This category includes food items designated for babies and toddlers that do not fall into the other subcategories. Includes products such as cheese for babies, individual sauces for pasta and separate pasta items, as well as dressings for babies. Excludes products intended to treat dehydration or medical conditions.

GNPD, Global New Products Database.

**Table A2:** Baby food products retrieved from the Mintel GNPD database for the period of March 2017–March 2021 by food category and country.

	Baby Cereals	Baby Biscuits and Rusks	Baby Juices and Drinks	Baby Fruit Products, Desserts and Yoghurts	Baby Snacks	Baby Savoury Meals and Dishes	Other Baby Food	Total
**Total**	**571**	**233**	**147**	**1306**	**394**	**721**	**55**	**3427**
Austria	31	14	7	55	20	25	1	153
Belgium	2	5	0	11	1	10	0	29
Bulgaria	0	1	0	0	0	0	0	1
Croatia	1	0	1	8	2	1	0	13
Czechia	10	12	11	67	15	31	0	146
Denmark	12	4	4	49	17	11	0	97
Estonia	0	1	0	0	0	0	1	2
Finland	15	3	2	30	13	8	0	71
France	40	34	6	153	12	125	3	373
Germany	118	38	51	221	75	105	7	615
Greece	1	3	1	3	4	0	0	12
Hungary	9	2	1	44	4	6	0	66
Ireland	0	2	2	3	3	3	0	13
Italy	17	14	6	34	3	28	17	119
Latvia	2	0	0	5	0	1	0	8
Lithuania	2	3	0	4	1	0	0	10
Netherlands	15	6	3	31	14	17	2	88
Poland	49	12	18	120	29	44	4	276
Portugal	7	6	1	33	5	2		54
Romania	8	9	1	4	1	0	3	26
Slovakia	15	7	13	39	8	2	3	87
Slovenia	0	0	1	4	5	1	0	11
Spain	61	22	7	152	3	77	0	322
Sweden	3	0	0	8	8	2	0	21
Norway	61	2	2	86	36	31	0	218
Switzerland	21	7	6	34	19	14	1	102
United Kingdom	71	26	3	108	96	177	13	494

GNPD, Global New Products Database.

## Appendix B

This appendix provides a series of boxplots that graphically present the distribution of energy content and nutrient density of the products examined by food category (Figure A1, Figure A2, Figure A3, Figure A4, Figure A5, Figure A6, Figure A7 and Figure A8).
